# Progress in Cancer

**Published:** 1903-09-05

**Authors:** 


### Progress in Cancer.

(*Concluded from page* 383.)

Meredith Powell[6] advocates the use of formalin for malignant tumours in strength from 1½ to 2 per cent. The malignant tissue is thrown off the healthy subjacent parts as a slough. If the solution used be weaker than 1½ per cent. there is not sufficient effect, and if greater than 2 per cent. the application is painful and only surface hardening takes place. He makes the application on absorbent lint, covering it with jaconet and cotton wool and bandaging. This dressing is changed six hourly. After the third or fourth dressing the odour and discharge cease. In three to seven days the tumour loses its elasticity and becomes darkened, friable and insensitive; becoming completely removed in 16–24 days. Formalin exerts a selective action on morbid tissue; it may be that the miniature more embryonic cells are less resistent than older tissues. This treatment is different from that advocated by McFeeley who recommended undiluted formalin as a caustic in rodent ulcers. Powell's method is practically painless to the patient and can be carried out without narcotics or anæsthetics of any kind. He has treated three cases in this way, a sarcoma of the breast, a scirrhus of the breast and a recurrent epithelioma of the lip.

G. Lenthal Cheatle[7] sees some connection between the development of the primary focus of cancer and the distribution of nerves and trophic areas. "There is reason" he says "for thinking that the incidence of cancer within a nerve area is not a fortuitous circumstance, but that it may be due to the direct or indirect nervous influence over that area." In the distribution of herpes, morphœa, moles, nævi and certain infective processes the particular nerves are sensory, but in carcinoma he is not prepared to say whether the following phenomena are due to the influence of peripheral nerves or whether they are due to a central nervous influence exhibiting itself in its peripheral nerve area. As he points out and shows with photographs, the spread of rodent ulcers takes place in the direction of these nerve areas—and when one area is involved there is some tendency not to overlap the contiguous area: thus a growth in the cervical spinal area does not tend to invade the contiguous fifth nerve area. Rodents, he has observed, as also Hutchinson's crateriform ulcer, and typical squamous epithelioma frequently arise at the points where nerves become cutaneous. Lugaro has proved that peripheral irritation causes central changes of degeneration in the posterior root ganglia of the spinal nerves. Irritation is a precursor of cancer, moles occur in nerve areas, cancer frequently commences in a mole; is there not some ætiological connection? Commenting on this suggestion of Cheatle's, Beatson[8] urges Goodsir's view of nutritive centres, or centres of germination. "Seeing," he says, "that the whole essence of the cancer process is cell proliferation, and that this latter can only develop in cells that have a special proliferating power, either acquired in one of several ways, or natural as in Cohnheim's 'rests' and Goodsir's 'centres of nutrition,' I think that these last as a possible explanation of the phenomena alluded to should be borne in mind."

**Pathology.**—Gaylord[9] has found evidence of nuclear division in the cell inclusions found in cancerous epithelium. The series of changes found in these "bird's eye" inclusions closely resemble the process of nuclear divisions described by Nauaschin in the amœboid form of plasmodiophora brassicæ, an organism known to produce tumours and outgrowths in plants. The spores of this latter organism when within the phagocytic cells of warm or cold blooded animals present an appearance indistinguishable from these inclusions under the same conditions of fixing and staining employed in the investigation of "bird's-eye" inclusions.

J. C. Hemmath[10] has confirmed Silbermann's statement that a permanent lesion can be obtained in a dog's stomach by denuding the mucosa near the pylorus and depriving the animal of hæmoglobin by means of the injection of pyrogallic acid. Such an ulcer can become carcinomatous spontaneously without efforts of inoculation. But four out of ten dogs so treated were successfully inoculated with carcinoma, and in another ten animals five developed carcinoma round the edges of the ulcer by the injection of a cell-free extract of carcinoma. He has noticed that in normal salt solution cancer cells swell up. This opens up a question as to whether the growth of cancer cells can be altered by external stimuli. It has been conceived that in a cell there are contained numerous smaller cells, each with a separate enzyme concerned in the synthesis of albumin or its destruction. A damage which causes a breakdown in the cell might produce an admixture of these enzymes and cause "anarchy in a previously orderly household." Enzymes are affected by heat and he has noticed that he cannot produce cancer artificially after heating the inoculation fluids up to 60°C. He refers to Virchow's three modes of cancer extension viz:—(1) immediate extension; (2) dissemination by metastasis; and (3) a transference by means of liquids which possessed the power of creating infection, thus causing the individual parts to form a reproduction of the same mass which was originally present. In this perhaps Virchow saw the possibility of the spread by means of the action of enzymes. It may be the serum contains a substance normally capable of destroying cancer cells, but not when these are present in inordinate quantities. If this be true our aim should be to augment the natural defence.

[6] Brit. Med. Jour., May 30. [7] Ibid., April 18. [8] Ibid., May 2. [9] Ibid., May 23. [10] Ibid., April 18.

